# Insulin dysfunction and Tau pathology

**DOI:** 10.3389/fncel.2014.00022

**Published:** 2014-02-11

**Authors:** Noura B. El Khoury, Maud Gratuze, Marie-Amélie Papon, Alexis Bretteville, Emmanuel Planel

**Affiliations:** ^1^Département de Psychiatrie et Neurosciences, Faculté de Médecine, Université LavalQuébec, QC, Canada; ^2^Axe Neurosciences, Centre Hospitalier de l'Université LavalQuébec, QC, Canada

**Keywords:** Alzheimer's disease, diabetes mellitus, Tau phosphorylation, kinases, phosphatases

## Abstract

The neuropathological hallmarks of Alzheimer's disease (AD) include senile plaques of β-amyloid (Aβ) peptides (a cleavage product of the Amyloid Precursor Protein, or APP) and neurofibrillary tangles (NFT) of hyperphosphorylated Tau protein assembled in paired helical filaments (PHF). NFT pathology is important since it correlates with the degree of cognitive impairment in AD. Only a small proportion of AD is due to genetic variants, whereas the large majority of cases (~99%) is late onset and sporadic in origin. The cause of sporadic AD is likely to be multifactorial, with external factors interacting with biological or genetic susceptibilities to accelerate the manifestation of the disease. Insulin dysfunction, manifested by diabetes mellitus (DM) might be such factor, as there is extensive data from epidemiological studies suggesting that DM is associated with an increased relative risk for AD. Type 1 diabetes (T1DM) and type 2 diabetes (T2DM) are known to affect multiple cognitive functions in patients. In this context, understanding the effects of diabetes on Tau pathogenesis is important since Tau pathology show a strong relationship to dementia in AD, and to memory loss in normal aging and mild cognitive impairment. Here, we reviewed preclinical studies that link insulin dysfunction to Tau protein pathogenesis, one of the major pathological hallmarks of AD. We found more than 30 studies reporting Tau phosphorylation in a mouse or rat model of insulin dysfunction. We also payed attention to potential sources of artifacts, such as hypothermia and anesthesia, that were demonstrated to results in Tau hyperphosphorylation and could major confounding experimental factors. We found that very few studies reported the temperature of the animals, and only a handful did not use anesthesia. Overall, most published studies showed that insulin dysfunction can promote Tau hyperphosphorylation and pathology, both directly and indirectly, through hypothermia.

## Introduction

Alzheimer's disease (AD) is a neurodegenerative disorder characterized by a progressive loss of memory and a decline of cognitive functions. With aging population as a main risk factor (Harman, 2002), this pathology becomes one of the most frequent neurodegenerative diseases and one of the top public health economic concerns since more than 35 million people worldwide were affected in 2010 (Querfurth and Laferla, 2010).

Amyloid plaques and neurofibrillary tangles (NFT) are the two histopathological hallmarks of AD. Amyloid plaques are composed of extracellular aggregates of the β-amyloid peptide (Aβ) (Glenner and Wong, 1984; Selkoe, 2001), while NFT are composed of abnormally phosphorylated Tau protein, assembled into paired helical filaments (PHF) (Brion et al., 1985; Grundke-Iqbal et al., 1986; Buée et al., 2000).

Only rare cases of early onset Familial Alzheimer's Disease (FAD) are caused by mutations in the amyloid precursor protein (APP) or presenilin genes. By contrast, the etiology of the vast majority of cases, which are sporadic late onset AD (LOAD), is still misunderstood due to its multifactorial components involving both genetic and environmental factors. Two other common syndromes in the elderly are associated with aging, i.e., diabetes mellitus (DM) and impaired glucose tolerance. It is estimated that, in 2030, the number of diabetic people over 64 years old will exceed 82 million cases in developing countries, and 48 million cases in developed countries (Wild et al., 2004).

To date, there is increasing evidence supporting a link between AD and insulin dysfunction (Frolich et al., 1999; Gasparini et al., 2002; Craft and Watson, 2004; De La Monte et al., 2009; Sims-Robinson et al., 2010). For example, compared to age-matched controls, AD brains show low concentrations of insulin accompanied by an increase in the number of insulin receptors (Frolich et al., 1999; Craft and Watson, 2004). Moreover, numerous population-based studies have examined the association between AD and insulin dysfunction. Some studies found no association (Arvanitakis et al., 2006; Thambisetty et al., 2013); while, interestingly, others suggest that hyperglycemia, impaired insulin secretion, glucose intolerance and insulin resistance are all associated with increased risk of AD (Luchsinger et al., 2004; Ronnemaa et al., 2008; Crane et al., 2013a). Thus, higher AD incidences rates were detected in elderly diabetic patients (Leibson et al., 1997; Ott et al., 1999; Luchsinger et al., 2004; Xu et al., 2004; Ronnemaa et al., 2008), and reduced mental skills have been observed in diabetic children (Northam et al., 2001; Schoenle et al., 2002; Fox et al., 2003; Dahlquist and Kallen, 2007; and for review, see Roriz-Filho et al., 2009). All this evidence has prompted the hypothesis that AD might be a form of brain diabetes (Hoyer, 1998; De La Monte et al., 2006).

In this article, we will review both *in vitro* and animal findings reporting a correlation between diabetes and Tau pathology, one of the major neuropathological hallmarks of AD. We will also focus on the mechanisms that might link insulin dysfunction to Tau pathology.

## Tau protein

### Structure and function

Tau is a microtubule-associated protein that is abundant in the central nervous system (CNS) and expressed mainly in axons. In the human brain, Tau proteins constitute a family of six isoforms, ranging from 352 to 441 amino acids, all derived from single gene by alternative splicing (Goedert et al., 1989). The amino-terminal region of Tau, also called the “projection domain,” project from the microtubule (MT) space to interact with the plasma membrane and is essential for determining axonal diameter (Chen et al., 1992). The carboxy-terminal region is characterized by the presence of 3 or 4 repeats that mediate the properties of Tau to stabilize MT and promote their polymerization (Weingarten et al., 1975; Cleveland et al., 1977a,b; Himmler et al., 1989; Butner and Kirschner, 1991; Gustke et al., 1994). These functions are negatively regulated by phosphorylation at multiple sites in, and around, the MT binding domain (for review, see Buée et al., 2000; Avila et al., 2004). Indeed, at least 30 Ser/Thr potential Tau phosphorylation sites have been described on Tau (Sergeant et al., 2008). Tau phosphorylation is regulated by numerous Ser/Thr kinases and phosphatases; among them glycogen synthase kinase-3β (GSK-3β) and protein phosphatase 2A (PP2A) are considered to be the major Tau kinase and phosphatase *in vivo*, respectively (Planel et al., 2002; Tian and Wang, 2002).

Intracellular aggregates of abnormally hyperphosphorylated Tau characterize a group of neurodegenerative diseases called “Tauopathies” (for review, see Buée et al., 2000). Tau hyperphosphorylation may lead to its aggregation *in vitro* (Alonso et al., 2001; Sato et al., 2002) and is thought to induce NFT formation and neurodegeneration in AD brains (Trojanowski and Lee, 1994).

### Interaction between Tau and amyloid pathology

The second major neuropathological hallmark of AD are amyloid plaques, mainly composed of amyloid-β (Aβ), a peptide of 39–43 amino acids generated *in vivo* by specific proteolytic cleavage of the APP, a transmembrane glycoprotein (Hardy and Higgins, 1992). It appears that soluble oligomeric Aβ forms, rather than amyloid plaques, contribute to the cellular pathology of the AD and correlate with the severity of cognitive impairment in humans (Lue et al., 1999; and for review, see Laferla et al., 2007).

To date, the interaction between Tau pathology and amyloid plaques in AD remains unresolved. Several murine animal models combining the two types of lesions have been investigated to better address this issue.

However, results have shown different possible types of interaction between the two pathologies. Thus, it was reported that injection of Aβ42 peptide potentiates Tau pathology in a mouse model of Tauopathy overexpressing a mutant form of the protein (Gotz et al., 2001). Similarly, double transgenic APP/Tau mice show early Tau pathology in comparison to Tau transgenic mice (Lewis et al., 2001; Perez et al., 2005; Ribe et al., 2005; Terwel et al., 2008). Interestingly, cultured neurons obtained from Tau^−/−^ mice seem to be protected against neuronal death and cytotoxicity induced by Aβ, suggesting that Tau is crucial for Aβ-induced neurotoxicity (Rapoport et al., 2002). These results were further supported *in vivo*, in animal studies combining both Tau^−/−^ and APP transgenic mice and showing that Tau reduction is protective against neurological and behavioral deficits induced by Aβ (Palop et al., 2006; Roberson et al., 2007). More importantly, this hypothesis is further supported by numerous *post-mortem* neuropathological studies showing that the pathway of Tau pathology is progressive, sequential and hierarchical (Bierer et al., 1995; Braak et al., 1999; Delacourte and Buée, 2000), whereas amyloid deposition is diffuse, widespread and extremely heterogeneous (Braak et al., 1999; Delacourte et al., 1999). Moreover, NFT formation was found to be strongly correlated with the degree of dementia and memory loss in AD brains, whereas Aβ was not (Braak and Braak, 1991, 1997; Delacourte et al., 1999). These observations have led some authors to the hypothesis that Tau pathology could be the major cause of cognitive decline in humans (Wilcock and Esiri, 1982; Delaere et al., 1989; Arriagada et al., 1992; Duyckaerts et al., 1997, 1998; Gomez-Isla et al., 1997; Delacourte et al., 2002; Giannakopoulos et al., 2003; Guillozet et al., 2003; Bretteville and Planel, 2008). These findings are in accordance with numerous animal studies showing significant impairment of cognitive functions, synaptic dysfunctions, as well as altered hippocampal synaptic plasticity in different transgenic mouse models of Tauopathies (Polydoro et al., 2009; Hoover et al., 2010; Van Der Jeugd et al., 2011; Burnouf et al., 2013).

## Insulin dysfunction

### Basics of insulin signaling

The insulin receptor (IR) is a hetero-tetrameric receptor composed of two extracellular α-subunits that bind insulin, and two transmembrane β-subunits that have intracellular tyrosine kinase activity. The binding of insulin to the α-subunits of IR leads to the activation of the β subunit via auto-phosphorylation. When activated, IR phosphorylates insulin receptor substrate (IRS) proteins, which then activate phosphoinositide-3 kinase (PI3K). Two major signaling pathways are further activated by PI3K: the mitogen-activated protein kinase (MAPK) pathway and the Akt pathway (for review, see White, 1997; Taha and Klip, 1999). MAPK pathway is mainly involved in cell differentiation, cell proliferation and cell death, whereas Akt signaling is implicated in cell proliferation, cell growth, and protein synthesis (for review, see Brazil and Hemmings, 2001; Le Roith and Zick, 2001; Tremblay and Giguere, 2008). Akt further induces the phosphorylation of GSK-3β, rendering it inactive, which results in increase of glycogen synthesis in the periphery. Thus, disruption of Akt signaling leads to the dephosphorylation of GSK-3β, and hence to its activation; (for review, see Taha and Klip, 1999).

In insulin resistance that occurs in T2DM, the capacity of insulin to induce all these phosphorylation cascades is significantly decreased. Muscle biopsies of insulin resistant subjects revealed a reduction in the enzymatic activity and phosphorylation state of kinases and substrates involved in the IR signaling pathway (for review, see Schulingkamp et al., 2000; White, 2003; Pirola et al., 2004; Youngren, 2007). However, the site(s) of the initial perturbation of signal transduction are far from clear.

### Insulin in the CNS

In the past, the brain was described as “an insulin insensitive organ” (Goodner and Berrie, 1977). However, it is currently well known that insulin and its receptors are widely present in the CNS (Baskin et al., 1988; Lerorrtt et al., 1988; for review, see Schulingkamp et al., 2000). Although the origin of brain insulin is still a matter of debate, several evidence suggest that central insulin is actively transported from periphery across the blood-brain barrier (BBB) (Banks et al., 1997a,b), and might be also produced locally by neurons in the brain (Devaskar et al., 1994).

The initial evidence of *de novo* insulin synthesis in the CNS came from a report showing insulin immunoreactivity in adult rat brain (Havrankova et al., 1978b). This evidence was later confirmed by other studies showing insulin gene expression and synthesis in cultured mammalian neurons (Schechter et al., 1988, 1990; Devaskar et al., 1994).

Interestingly, it was shown that high peripheral insulin concentrations acutely increase the levels of insulin in the brain and the cerebrospinal fluid (CSF), whereas prolonged peripheral hyperinsulinemia leads to a down-regulation of IRs in the BBB and to a reduction in insulin transport into the brain (Wallum et al., 1987).

IRs are selectively distributed in the brain. Thus, IRs mRNA levels are highly concentrated in several brain areas including the olfactory bulb, pyriform cortex, amygdala, hippocampus, hypothalamus and the cerebellar cortex (Marks et al., 1991; Zhao et al., 1999). Accordingly, immunohistochemistry studies revealed that IRs are highly expressed in the olfactory bulb, hypothalamus, cerebral cortex, amygdala and hippocampus (Havrankova et al., 1978a,b; Baskin et al., 1987; Marks and Eastman, 1990; Marks et al., 1991; Unger et al., 1991; Zhao et al., 1999). Differences between peripheral and central IRs are not well understood, although some structural differences, mainly including the molecular weight and antigenicity, have been noted (Heidenreich et al., 1983; Heidenreich and Gilmore, 1985).

The expression of IRs in specific brain regions, particularly the hippocampus and medial temporal cortex suggests that insulin is implicated in memory processes (Singh et al., 1997; Zhao et al., 1999; Duarte et al., 2012; De Felice, 2013). For example, it was reported that both mRNA and protein levels of IRs are increased in the rat brain following a spatial memory task, suggesting that insulin might regulate normal memory function (Zhao et al., 1999).

By contrast, several findings have showed that insulin dysfunction, e.g., chronic hyperinsulinemia, DM or insulin resistance have a negative impact on memory process and cognitive function (Roriz-Filho et al., 2009; Sims-Robinson et al., 2010; Benedict et al., 2012; Crane et al., 2013b).

Mechanisms by which insulin might influence memory are reviewed elsewhere (Zhao and Alkon, 2001; Craft and Watson, 2004; Gerozissis, 2008; Freiherr et al., 2013). In brief, insulin signaling was shown to regulate neuronal survival, neurotransmission and synaptic activities (Zhao and Alkon, 2001). Insulin might also regulate synaptic plasticity by modulating long-term potentiation (LTP) (Zhao et al., 2010; Nistico et al., 2012), and promoting long-term depression (LTD) (Van Der Heide et al., 2005; Labouèbe et al., 2013), two major mechanisms involved in learning and memory.

In this context, it was demonstrated that treatment with insulin sensitizers (e.g., liragultide, pioglitazone) ameliorates central insulin resistance (Han et al., 2013; Hu et al., 2013; Yang et al., 2013), improves cognitive impairment (Han et al., 2013), enhances synaptic plasticity (Han et al., 2013), and significantly decreases levels of Tau phosphorylation in the rat brain (Hu et al., 2013; Yang et al., 2013).

Interestingly, it was recently established that administration of intranasal insulin is a non-invasive technique that leads to an increase of insulin levels specifically in the CNS, without affecting peripheral glucose and insulin levels (Reger et al., 2008a). Thus, several clinical studies have demonstrated a beneficial effect of intranasal insulin on memory and cognitive function in AD patients (Reger et al., 2008a,b; Craft et al., 2012).

Therefore, although emerging evidence support a role of brain insulin signaling in several complex CNS functions, additional mechanistic studies are warranted to better elucidate the contribution of insulin signaling in normal and diseased brains.

### Diabetes mellitus

Diabetes mellitus (DM) is a chronic disease, marked by high levels of blood glucose. It occurs in two main forms: type 1 and type 2 diabetes, that respectively represent about 5–10% and 90–95%, of the total diabetes cases (CDC, 2008).

#### Type 1 diabetes mellitus

Type 1 diabetes mellitus (T1DM) was previously known as Insulin-dependent DM (IDDM) or juvenile diabetes to distinguish it from type 2 diabetes mellitus (T2DM), which generally has a later onset; however, the majority of new-onset type 1 diabetes is seen in adults (Naik and Palmer, 2007).

It is due to autoimmune destruction of the insulin-producing β-cells resulting in low or no production of insulin hormone. Thus, insulin therapy is absolutely required in patients with T1DM (McCrimmon and Sherwin, 2010). However, one major feared side effect of insulin therapy is iatrogenic hypoglycemia, which might occur in almost 90% of all insulin treated patients (Cryer, 1997, 2001, 2004; Cryer et al., 2003).

#### Type 2 diabetes mellitus

Type 2 diabetes mellitus (T2DM), previously known as non-insulin dependent DM (NIDDM), was usually associated with aging. About 40% of the population over 65 and 50% over 80 years of age have T2DM or impaired glucose tolerance, and half of diabetic people are not diagnosed (Lamberts et al., 1997). However, T2DM is not a disease exclusively associated with old age, as it is more and more diagnosed in young people as the number of cases has risen in parallel with obesity. Today, there are 382 million people with T2DM, and it is estimated that this number will rise to 592 million by 2035 (IDF, 2013). In T2DM, pancreas produces insulin, but, for reasons that remain unclear, cells are unable to use it, a characteristic of insulin resistance etiology. Thus, in a positive feedback loop, pancreas overproduces insulin to overcome the resistance, leading to a hyperinsulinemia in early phases of T2DM (Festa et al., 2006). Over time, the suffering pancreas becomes unable to produce enough insulin, leading to hyperglycemia and insulin deficiency that accompany T2DM in the end of its natural history (Defronzo, 2004).

## Impact of insulin dysfunction on Tau phosphorylation

Over the last decade, there has been considerable interest on the impact of insulin dysfunction and diabetes on Tau pathology. It has been shown that insulin could regulate Tau phosphorylation in neuronal cells (Hong and Lee, 1997; Lesort et al., 1999; Lesort and Johnson, 2000), which was confirmed by observations of hyperphosphorylated Tau in mice showing abnormal insulin levels in the brain (Schubert et al., 2003, 2004; Planel et al., 2004; Schechter et al., 2005). Despite all these data, very little is known about the effects of diabetes on Tau pathogenesis *in vivo*.

### *In vitro* studies

Observations from *in vitro* studies strongly suggest that Tau phosphorylation might be regulated by insulin signaling through a biphasic manner. Thus, short insulin treatment (<2–3 min), either in rat primary cortical neurons (Lesort and Johnson, 2000), or in SH-SY5Y human neuroblastoma cells (Lesort et al., 1999), leads to a rapid and transient Tau hyperphosphorylation at the AT8 (Ser^202^/Thr^205^), AT180 (Thr^231^), PHF-1 (Ser^396^/Ser^404^) and T3P (Ser^396^) epitopes (Lesort et al., 1999; Lesort and Johnson, 2000). By contrast, prolonged exposure to insulin, in both human neuroblastoma cells (up to 60 min of treatment) (Lesort et al., 1999), and human neuronal NT2N cells (5 min of treatment) (Hong and Lee, 1997), results in a significant decrease in Tau phosphorylation. Interestingly, all these studies have led to the conclusion that the effects of insulin are mediated by GSK-3β activity (Hong and Lee, 1997; Lesort et al., 1999; Lesort and Johnson, 2000).

Therefore, insulin signaling plays an important role in the regulation of Tau phosphorylation, and this effect is correlated with GSK-3β activity, further suggesting a key role of this kinase in Tau pathology.

### *In vivo* studies

The specific effects of insulin dysfunction on Tau pathology are not fully understood. Therefore, several animal models are currently used to investigate the impact of both T1DM and T2DM on Tau phosphorylation.

#### T1DM and Tau phosphorylation

Models of T1DM used in the literature mainly mimic insulin deficiency that characterizes human T1DM. These models can develop T1DM either spontaneously, due to a specific genetic background, or by treatment with a drug called Streptozotocin (STZ).

STZ (2-deoxy-2-(3-(methyl-3-nitrosoureido)-D-glucopyr-anose)) is a toxin synthesized by *Streptomycetes achromogenes*, that specifically enters pancreatic β cells via the glucose transporter GLUT2, and leads to cell death through DNA alkylation (Delaney et al., 1995). Thus, it is widely used to induce insulin deficiency associated to T1DM (Ganda et al., 1976), both in wild-type background context and AD pathological animal models.

***STZ-induced wild-type animal models***. Using an extensive panel of phospho-dependent antibodies that detect specific epitopes on Tau protein, several animal studies have investigated the impact of peripheral STZ treatment on Tau phosphorylation in non-transgenic animals (Table 1). For example, we have previously reported that STZ administration (200 mg/Kg, i.p.) resulted in a massive Tau hyperphosphorylation at the Tau-1 (Ser^195^/Ser^198^/Ser^199^/Ser^202^), pS199 (Ser^199^), PHF-1, AT8, AT180, Ser^422^, Ser^262^, Ser^356^ and Ser^400^ epitopes in the brain of non-transgenic mice (Planel et al., 2007b). Other studies have confirmed our results of increased Tau phosphorylation following STZ treatment either in mice (Clodfelder-Miller et al., 2006; Jolivalt et al., 2008; Ke et al., 2009; Kim et al., 2009), or in rats (Qu et al., 2011), and observed a further increase in Tau phosphorylation at the AT270 (Thr^181^) and Thr^212^ epitopes (Table 1).

**Table 1:** **Summary of animal studies showing Tau hyperphosphorylation and its molecular mechanisms in diabetic animal models**.

**References**	**Animal model**	**Diabetes context**	**Tau phosphorylation sites**	**Tau kinases**	**Tau PP**	**Insulin signaling**	**Anesthesia prior to sacrifice**	**T(°C)**
	**T1DM-WT**	**STZ-induced (i.p.)**						
Clodfelder-Miller et al., 2006	C57BL6/J (M)	150 mg/Kg	AT8, AT180, AT270, PHF-1, 12E8, S199, T212 (+), Tau-1 (−)	pJNK, p38 (+), pS9 GSK3β (+)	PP2A (−)	N.D.	N.R.	No
Planel et al., 2007b	C57BL6/J (M)	200 mg/Kg	AT8, AT180, PHF-1, S199, S422, S262, S356, S400 (+), Tau-1 (−)	pCamKII, pJNK (+), pS9 GSK3β (+)	PP2A (−)	N.D.	No	Yes
Kim et al., 2009	C57BL6/J (M)	150 mg/Kg	T231, S199, 202, 396 (+)	N.D.	N.D.	N.D.	Yes	No
Jolivalt et al., 2008	Swiss-Webster (M)	90 mg/Kg	T231, PHF-1 (+)	pS9 GSK3β (−), pGSK3α (−), pAkt (−)	N.D.	pIR, PDK1 (−)	Yes	No
Qu et al., 2011	Sprague-Dawley (R)	55 mg/Kg	PHF-1 (+), Tau-1 (−)	pS9 GSK3β (−), pAkt (−)	PP2A (−)	N.D.	N.R.	No
	**T1DM-Tg**	**STZ-induced (i.p.)**						
Jolivalt et al., 2010	hAPP (M)	90 mg/Kg	T231, AT8, PHF-1 (+)	pS9 GSK3β (−), pY216 GSK3β (+)	N.D.	pIR (−)	Yes	No
Ke et al., 2009	pR5 (M)	200 mg/Kg	AT8, AT100, AT270, 12E8, PHF-1, S422 (+)	N.D.	N.D.	N.D.	N.R.	No
	**T1DM**	**Genetic**						
Li et al., 2007	BB/Wor (R)	Spontaneous	S396 (0)	pS9 GSK3β (−), pAkt (−)	N.D.	IRβ (−)	Yes	No
Papon et al., 2013	NOD (M)	Spontaneous	AT8, CP13, TG3, S422, 262, PHF-1 (+), Tau-1 (−)	pAkt (+), pS9 GSK3β (+), pCamKII, Cdk5, p35 (−)	PP2A, PP2B (−), PP5 (+)	N.D.	No	Yes
Schechter et al., 2005	Insulin^−/−^ (M)	KO	AT180 (+)	pJNK (+), pS9 GSK3β (+), pMAPK (−)	N.D.	N.D.	N.R.	No
	**T2DM-WT**	**HFD-induced (Kcal%)**						
Moroz et al., 2008	C57BL6/J (M)	60%	N.D.	N.D.	N.D.	IRS-1, IRS-4 ARNm (+)	No	No
Becker et al., 2012	C57BL/ 6NTac (M)	~25%	AT8, AT180, AT270, S199, 396 (0)	pAkt, pMAPK, pJNK, Cdk5, pS9 GSK3β (0)	PP2A (0)	N.D.	N.R.	No
	**T2DM-WT**	**HFC-induced**						
Bhat and Thirumangalakudi, 2013	C57BL6/J (M)	21% Fat, 1.25% Cholesterol	PHF-1, T231 (+)	pS9 GSK3β (−), pAkt (−)	N.D.	IRS-1 (−)	Yes	No
	**T2DM-Tg**	**HFD-induced (Kcal%)**						
Julien et al., 2010	3xTg-AD (M)	60%	PHF1, CP13, AT270 (0)	N.D.	N.D.	N.D.	Yes	No
Leboucher et al., 2012	THY-Tau22 (M)	59%	S202, T205, S214, S404, S422 (+)	pAkt (+), pS9 GSK3β (+)	PP2A (0)	IRS1 (+)	N.R.	No
	**T2DM**	**Genetic**						
Jung et al., 2013	OLETF (R)	Spontaneous	T212 (+), T231 (+), S262 (+), S396 (+)	pS9 GSK3β (0), Cdk5 (0), CamKII (+)	PP2A (−)	N.D.	Yes	No
Li et al., 2007	BBZDR/ Wor (R)	Spontaneous	S396 (+)	pS9 GSK3β (−), pAkt (−)	N.D.	IRβ (0)	Yes	No
Jung et al., 2011	OLETF (R)	Spontaneous	T212 (+), Tau-1 (−)	pS9 GSK3β, pY216 GSK3β, Cdk5 (0)	PP2A, PP2B (0)	IRβ (+)	Yes	No
Kim et al., 2009	db/db (M)	Spontaneous	AT8, S199, 202, 396, 422 (+)	N.D.	N.D.	N.D.	Yes	No
Li et al., 2012a	db/db (M)	Spontaneous	S396 (+)	pJNK (+)	PP2A (−)	N.D.	Yes	No
Schubert et al., 2004	NIRKO (M)	KO	AT180 (+)	pS9 GSK3β (−), pAkt (−)	N.D.	N.D.	N.R.	No
Schubert et al., 2003	IRS-2^−/−^ (M)	KO	AT8 (+)	pS9 GSK3β (+)	PP2A (−)	IRβ, pIR, IRS-1, IRS-2 (−)	N.R.	No
	**T3DM-WT**	**STZ-induced (i.c.v.)**						
Salkovic-Petrisic et al., 2006	Wistar (R)	1 mg/Kg	N.D.	p-GSK3α/β (+), Akt (−)	N.D.	N.D.	N.R.	No
Grunblatt et al., 2007	Wistar (R)	1 mg/Kg	PHF-1 (+)	N.D.	N.D.	pRI (+), IRβ (−)	N.R.	No
Deng et al., 2009	Wistar (R)	1.5 mg/Kg	PHF-1, S199, 396, T212 (+)	pS9 GSK3β (−), pMAPK (−)	N.D.	pPI3K (−)	No	No
Chu and Qian, 2005	Sprague-Dawley (R)	3 mg/Kg	S202, 396, 404 (+)	N.D.	N.D.	N.D.	N.R.	No
Chen et al., 2011	Wistar (R)	3 mg/Kg	S396, T181 (+)	pS9 GSK3β (−), pY216 GSK3β (+), pAkt (−)	N.D.	N.D.	N.R.	No
Li et al., 2012b	Wistar (R)	3 mg/Kg	AT8 (+)	N.D.	N.D.	N.D.	N.R.	No
Chen et al., 2013a	C57BL6/J (M)	3 mg/Kg	S199/202, T205 (+), S214 (−)	pS9 GSK3β (0), pAkt (0)	N.D.	IRS-1, p-IRS-1, PI3K, p-PI3K (+), p-PDK1 (−)	N.R.	No
Du et al., 2013	Sprague-Dawley (R)	3 mg/Kg	T205 (+), S396 (+), Tau-1 (−)	pS9 GSK3β (0), pMAPK (0), pJNK (0)	p-PP2AC (0)	N.D.	N.R.	No
	**T3DM-Tg**	**STZ-induced (i.c.v.)**						
Plaschke et al., 2010	Tg2576 (M)	1.25 mg/Kg	AT8 (0)	pGSK3-α/β (−)	N.D.	N.D.	Yes	No
Chen et al., 2013b	3xTg-AD (M)	3 mg/Kg	S199/S202 (+), 12E8 (+), S422 (+)	GSK3β (−), pS9 GSK3β (−), GSK3α (−), pGSK3α (+), Akt (−), pAkt (+)	N.D.	IRβ (+), PI3K (+), PDK-1 (−)	N.R.	No

*T1DM: Type 1 diabetes mellitus; T2DM: Type 2 diabetes mellitus; T3DM: Type 3 diabetes mellitus; T(°C), temperature; WT, wild-type; Tg, transgenic; M, mouse; R, rat; STZ, streptozotocine; i.p., intra-peritoneal; i.c.v., intra-cerebroventricular; HFD, high-fat diet; hAPP, human Amyloid precursor protein; pR5, human Tau trangenic P301L (pR5 construct); BB/Wor, BioBreeding/Worcester; NOD, non-obese diabetic; BBZDR/Wor, bio-breeding Zucker diabetic rat/Worcester; OLETF, Otsuka long evans Tokushima fatty; KO, knockout; NIRKO, neuronal insulin receptor knockout; 3xTg-AD, triple transgenic Alzheimer's disease; pJNK, phospho-c-Jun N-terminal kinase; pMAPK, phospho-mitogen-activated protein kinase; GSK-3β, glycogen synthase kinase 3β; pS9 GSK-3β, phospho-Serine 9 GSK-3β, it is an inhibitory phosphorylation; pCamkII, phospho-Ca2+/calmodulin-dependent protein kinase; Cdk5, cyclin-dependent kinase 5; PP, protein phosphatase; PP2AC, catalytic subunit of PP2A; IR, insulin receptor; IRβ, β subunit of IR; PDK1, 3-phospho-inositide-dependent protein kinase-1; IRS, insulin receptor substrate; pPI3K, phospho-phosphatidylinositol 3-kinase; (−), decrease; (+), increase; (0), non-significant, N.D., non-determined; N.R., not reported*.

Some of the phosphoepitopes detected are associated to particular functions during Tau pathology. For example, Ser^422^ is associated with early pre-tangle formation and is characteristic of abnormal, AD-like Tau phosphorylation (Augustinack et al., 2002). AT8 is considered an early marker of Tau dysfunction (Matsuo et al., 1994; Hasegawa et al., 1996; Bussière et al., 1999; Augustinack et al., 2002), whereas PHF-1 is associated with late paired helical filament and NFT formation (Bramblett et al., 1993; Goedert et al., 1993, 1994). In addition, some phosphorylation sites have been linked to specific aspects of Tau pathology such as the sequestration of normal Tau (e.g., Thr^231^/Ser^235^) (Alonso et al., 1997, 2004), the inhibition of Tau MT binding (e.g., Ser^262^) (Biernat et al., 1993; Drewes et al., 1995), and the promotion of Tau aggregation (e.g., Ser^396^, Ser^422^) (Gong and Iqbal, 2008).

Interestingly, we have shown that the massive Tau hyperphosphorylation in STZ treated mice was mostly, but not completely, rescued by returning animals to normothermia, suggesting that hypothermia was partially involved in STZ-mediated Tau hyperphosphorylation. We have previously demonstrated that Tau phosphorylation can be induced by hypothermia (Planel et al., 2004, 2007b). Moreover, we have shown that this mechanism is mainly attributable to a direct and rapid inhibition of PP2A activity in the brain (Planel et al., 2004).

In fact, hypothermia is a common outcome in experimental diabetes (Shalaby et al., 1989; Kilgour and Williams, 1996, 1998) and several human diabetic populations (Neil et al., 1986; Scott et al., 1987).

In addition, Tau phosphorylation is known to be exquisitely sensitive to temperature, increasing by 80% per degree Celsius drop under 37°C in mice (Planel et al., 2007a; Papon et al., 2011). It has to be mentioned that previous studies done in other laboratories described above did not monitor the temperature of animals and therefore it is likely that hypothermia could contribute to the observed Tau hyperphosphorylation in these studies (Clodfelder-Miller et al., 2006; Ke et al., 2009; Kim et al., 2009; Qu et al., 2011).

Therefore, temperature control during Tau analysis experiments in animal models is crucial to avoid artifactual hypothermic Tau hyperphosphorylation.

***STZ-induced transgenic animal models***. Beside studies performed in a physiological context, several investigators have addressed the impact of STZ treatment on Tau phosphorylation and NFT formation in transgenic mouse models of AD (Table 1). For example, Ke et al. have shown that insulin depletion increases more significantly Tau phosphorylation at multiple epitopes in mice that overexpress P301L mutant human Tau, and are prone to develop NFT (Gotz et al., 2001). This suggests that experimental diabetes leads to advanced NFT formation, as well as early neurofibrillar deposition in Tau transgenic mice (Ke et al., 2009). Similarly, insulin deficiency enhances the severity of Tau phosphorylation in the hippocampi of APP transgenic mice, which are characterized by the accumulation of the β-amyloid peptide and high levels of Aβ-immunoreactive plaques (Jolivalt et al., 2010).

Therefore, it seems that STZ-induced T1DM recapitulate NFT formation observed during of AD, and this effect is further worsened when the induction is combined to genetic pre-disposition to AD.

However, it is important to note that several studies from those cited above have used anesthesia prior to sacrifice (Clodfelder-Miller et al., 2006; Jolivalt et al., 2008, 2010; Ke et al., 2009; Kim et al., 2009) (Table 1), which is known to enhance Tau phosphorylation (Planel et al., 2007a; Papon et al., 2011; Whittington et al., 2011; Le Freche et al., 2012). This increase can be induced either directly, through a pharmacological effect of the drug itself (Whittington et al., 2011; Le Freche et al., 2012), or indirectly, through anesthesia-induced hypothermia (Planel et al., 2007a).

Thus, further animal studies with temperature control are warranted to separate the impact of hypothermia from that of insulin dysfunction on Tau phosphorylation.

***Animal models of spontaneous T1DM***. Although several studies have reported Tau hyperphosphorylation in STZ-induced animal models, it is important to extend these findings to a model that does not require a drug to induce T1DM, because, as we will see later, if STZ reaches the brain in minute quantities, it can lead to Tau hyperphosphorylation on its own.

Thus, we have investigated Tau hyperphosphorylation in the non-obese diabetic (NOD) mouse, one of the most valuable genetic animal models for T1DM (Leiter, 2001). These mice spontaneously develop T1DM at 13–25 weeks of age as a consequence of selective destruction of insulin-producing β cells (Makino et al., 1980). Interestingly, our data indicated that Tau hyperphosphorylation correlates with the appearance of spontaneous diabetes in adult NOD mice, and this effect was exacerbated when the mice became hypothermic as a consequence of diabetes. Interestingly, even in the absence of any deregulation in the glucose metabolism, we have observed a slight increase in Tau phosphorylation at Tau-1 and Ser^422^ epitopes in non-diabetic adult NOD mice. Moreover, the onset of diabetes (the appearance of hyperglycemia and glycosuria) was correlated with an extensive Tau hyperphosphorylation at the AT8, CP13 (Ser^202^), Ser^262^ and Ser^422^ epitopes in comparison to control. Furthermore, the appearance of hypothermia further extended Tau hyperphosphorylation to PHF-1 and TG3 (Thr^231^) epitopes in diabetic NOD mice (Papon et al., 2013), (Table 1).

Of note, Li et al. have also investigated Tau phosphorylation in BB/Wor rats, another model of spontaneous T1DM. Although these authors did not find any significant changes in the levels of Tau phosphorylation at the Ser^396^ epitope (Li et al., 2007), it should be mentioned that there was no report of the rats temperature and that they were anesthetized, which could have masked a potential elevation in Tau phosphorylation.

Thus, while our study demonstrated Tau hyperphosphorylation in NOD mice, additional studies should be done to our results in other animal models of spontaneous T1DM.

***The insulin knockout animal model***. In addition to genetic and STZ-induced animal models, the insulin knockout mouse model (Insulin^−/−^) might also mimic insulin deficiency that characterizes T1DM. These mice rapidly develop DM, show a dramatic glycosuria, and die within 48 h (Duvillie et al., 1997).

Interestingly, Schechter and colleagues have investigated the impact of insulin deficiency on Tau phosphorylation in Insulin^−/−^ mice. Thus, among all phospho-epitopes investigated, these authors have observed a significant increase in Tau phosphorylation at the AT180 epitope, suggesting that insulin signaling might affect Tau phosphorylation *in vivo* (Schechter et al., 2005), (Table 1).

#### T2DM and Tau phosphorylation

Models of T2DM used in animal studies mainly reproduce human T2DM features, including obesity, hyperglycemia, hyperinsulinemia, and insulin resistance. These models develop T2DM either spontaneously, or by using a variety of treatments including special diets.

***Animal models of spontaneous T2DM***. Several genetic animal models are used in the literature to address the impact of genetic T2DM on AD pathogenesis (Ellis et al., 1998; Li et al., 2007; Jolivalt et al., 2008; Takeda et al., 2010). These models are mainly characterized by an impairment of leptin signaling, by either lacking leptin (ob/ob mice) (Coleman, 1978), or carrying specific mutations in the leptin receptor (Bio-Breeding Zucker Diabetic Rats/Wor (BBZDR/Wor) rats and db/db mice) (Hummel et al., 1966; Tirabassi et al., 2004). Leptin is an adipocyte-specific hormone that plays important role in satiety and energy expenditure. It acts through the leptin receptor, a single-transmembrane-domain receptor of the cytokine receptor family. However, mechanisms by which leptin signaling leads to the development of T2DM are not well understood.

Several studies have reported Tau hyperphosphorylation in rat (Li et al., 2007; Jung et al., 2011, 2013) and mouse models (Kim et al., 2009; Li et al., 2012a) of spontaneous T2DM. For example, Jung et al. have reported an increase in Tau phosphorylation at the Tau-1, Thr^212^ (Jung et al., 2011, 2013), Thr^231^, Ser^262^ and Ser^396^ (Jung et al., 2013) epitopes in the chronic Otsuka Long Evans Tokushima Fatty (OLETF) T2DM rat model, and these changes were further increased with age (Jung et al., 2013). In addition, Li and colleagues have also observed Tau hyperphosphorylation at the Ser^396^ site in the cortex of BBZDR/Wor rat strain (Li et al., 2007). These results were also confirmed in db/db mice that showed Tau hyperphosphorylation at the AT8, Ser^199^, Ser^202^, Ser^396^, and Ser^422^ epitopes (Kim et al., 2009; Li et al., 2012a) (Table 1). However, since these animals were anesthetized prior to sacrifice and their temperature was not reported, it would be interesting to repeat these experiments in controlled conditions.

***Animal models of induced T2DM***. High fat diet (HFD) is commonly used to induce T2DM in animals. Moroz et al. have reported that HFD increases body weight, reduces brain weight and leads to brain insulin resistance, since mice fed with HFD showed increased brain insulin gene expression paralleled with decreased IR binding (Moroz et al., 2008). However, levels of both phosphorylated and total Tau were not affected in the brain of non-transgenic animals treated with HFD, although Tau mRNA levels were significantly increased (Moroz et al., 2008). Similarly, levels of total soluble and insoluble Tau, but not phospho-Tau were increased in HFD fed 3xTg-AD transgenic mice, suggesting that effects of HFD are modest on Tau phosphorylation (Julien et al., 2010).

On the other hand, a recent study has demonstrated that HFD leads to Tau phosphorylation in a model of Tauopathy treated with HFD, in a manner independent of insulin resistance, suggesting that, other obesity-related factors, might contribute to Tau pathology (Leboucher et al., 2012).

Interestingly, Bhat and Thirumangalakudi have reported that feeding mice with high-fat/cholesterol (HFC) diet also leads to peripheral insulin resistance. Importantly, these authors showed that HFC results in increased levels of Tau hyperphosphorylation at the PHF-1 and Thr^231^ epitopes in the mice hippocampi (Bhat and Thirumangalakudi, 2013).

It is difficult to draw conclusion from HFD treatments since published results are so divergent. The reasons for these differences could include the composition of the diet, the duration of the treatment, the background strains, the levels of contamination of the animal facilities and, of course, confounding hypothermia.

***Insulin signaling impairment animal models***. The group of Schubert et al. have generated and explored a brain/neuron insulin receptor knockout (NIRKO) mouse model that exhibit a conditional inactivation of the IR gene in the CNS (Schubert et al., 2004). These mice are mainly characterized by hyperinsulinemia, mild insulin resistance, obesity and reduced fertility (Bruning et al., 2000), and might therefore represent a model of T2DM. Central IR deficiency in these mice has resulted in a significant increase of Tau phosphorylation at the AT180 epitope (Schubert et al., 2004).

Another model of insulin signaling impairment that might represent a model of T2DM is the IRS-2^−/−^ mouse model. These mice are insulin resistant and develop diabetes as a result of impairment in survival and function of pancreatic β-cells (Bruning et al., 1998; Withers et al., 1998, 1999; Kulkarni et al., 1999; Michael et al., 2000). Schubert et al. have demonstrated that IRS-2^−/−^ mice exhibit Tau hyperphosphorylation at the Ser^202^ epitope (Schubert et al., 2003), (Table 1).

Therefore, animal models of insulin signaling dysfunction have provided additional evidence that insulin plays crucial role in the regulation of Tau phosphorylation *in vivo*. However, further studies are needed to better understand mechanisms underlying these effects.

#### T3DM and Tau phosphorylation

Data from T1DM and T2DM animal models strongly suggest that insulin signaling plays a key role in modulating AD pathogenesis. However, in almost all animal models cited above, Tau pathology was investigated in a context of peripheral insulin dysfunction. Therefore, animal models exhibiting specific alteration of the central insulin function might be helpful to address the role of brain insulin disruption in AD pathology. Thus, the group of Hoyer and colleagues have suggested that rats treated with intra-cerebroventricular (i.c.v.) STZ represent a sporadic AD animal model characterized by a specific insulin-resistant brain state (Nitsch and Hoyer, 1991; Duelli et al., 1994; Lannert and Hoyer, 1998; Salkovic-Petrisic and Hoyer, 2007). Interestingly, de la Monte et al have later proposed that AD might represent a specific form of brain diabetes and thus proposed the term of “type 3 diabetes” (T3DM) (De La Monte et al., 2006; Lester-Coll et al., 2006). Importantly, this hypothesis is supported by a recent *postmortem* study demonstrating a state of brain insulin resistance in human AD patients (Talbot et al., 2012).

In induced T1DM animal models, STZ is administrated peripherally at high doses (55–200 mg/Kg). However, in T3DM rats, STZ is injected in the brain ventricles at doses of up to 100 times lower than those used in systemic injections (Nitsch and Hoyer, 1991; Duelli et al., 1994; Lannert and Hoyer, 1998). Although it was shown that pancreatic architecture, as well as levels of both insulin and glucose are not affected following central STZ treatment (De La Monte et al., 2006; Lester-Coll et al., 2006), numerous studies have reported that T3DM rat models exhibit several neurochemical, structural and behavioral changes that are similar to cellular abnormalities observed in AD brains. These changes include brain atrophy, cell loss, neurodegeneration (De La Monte et al., 2006; Lester-Coll et al., 2006), a decrease in glucose utilization notably in the hippocampi and entorhinal cortices of treated rats (Duelli et al., 1994), a reduction of energy metabolism (Lannert and Hoyer, 1998; Prickaerts et al., 2000; Yun et al., 2000), an impairment in learning and memory, as well as a significant increase in oxidative stress (Sharma and Gupta, 2001; Veerendra Kumar and Gupta, 2003; Ishrat et al., 2006; Pathan et al., 2006).

The mechanism of action of central STZ is far from clear, but some observations suggest that it might be similar to that in the periphery. Indeed, GLUT2, the glucose transporter targeted by peripheral STZ, was found to be regionally distributed in the mammalian brain (Brant et al., 1993; Leloup et al., 1994; Ngarmukos et al., 2001). In addition, DNA damage was observed in T3DM rat brains (Nitsch and Hoyer, 1991; Lannert and Hoyer, 1998).

The vast majority of studies that have investigated Tau phosphorylation following i.c.v. STZ administration (ranging from 1 to 3 mg/Kg) were performed in non-transgenic animal models. Interestingly, all these studies have observed a significant increase in Tau phosphorylation at several epitopes, notably AT8, PHF-1, Ser^199^, Ser^202^, Ser^396^, Ser^404^, Thr^181^, Thr^205^, Thr^212^, and Tau-1(Chu and Qian, 2005; Salkovic-Petrisic et al., 2006; Grunblatt et al., 2007; Deng et al., 2009; Chen et al., 2011, 2013b; Li et al., 2012b; Du et al., 2013) (Table 1).

Interestingly, Chen et al. have recently investigated the impact of T3DM on Tau phosphorylation in the 3xTg-AD mouse model. These authors demonstrated an increase in Tau phosphorylation at the Ser^199^/Ser^202^, 12E8 (Ser^262^/Ser^356^) and Ser^422^epitopes (Chen et al., 2013b).

By contrast, Plascke et al. didn't detect any increase in the level of phosphorylated Tau in the brain of Tg2576 mice (Plaschke et al., 2010).

Therefore, T3DM leads to several alterations resembling to those found in human AD patients, further giving consistent evidence that brain insulin resistance might be a central event in AD. However, it should be noted that it remain difficult to conclude from all studies cited above because they are quite heterogeneous, considering differences in the rat strain used, the dose of STZ delivered, the age at STZ treatment and brain regions investigated (for review, see Salkovic-Petrisic et al., 2006).

## Mechanisms linking insulin dysfunction to Tau pathology

### Kinases activation

The increased phosphorylation of Tau on many residues might be attributable to the activation of multiple kinases. Among the kinases able to phosphorylate Tau *in vitro*, GSK-3β is considered to be the major physiological and pathological Tau kinases (Planel et al., 2002; Hernandez et al., 2013).

Studies that have addressed GSK-3β activation in diabetic animal models (using antibodies detecting the phosphorylation state either at the Ser9 inhibitory site, or the Tyr216 activation site of the kinase) have shown contradictory findings (Table 1), thus making it difficult to conclude about the implication of this kinase in the correlation between diabetes and Tau pathogenesis. Of note, we (Planel et al., 2007b), and others (Clodfelder-Miller et al., 2006; Deng et al., 2009), have demonstrated that GSK-3β is inhibited in STZ-induced T1DM mice, and our data demonstrated that this inhibition can be attributed to hypothermia (Planel et al., 2001), as GSK-3β S9 phosphorylation is a constant feature of hypothermia in mouse brain during metabolic stress (Planel et al., 2004) or anesthesia (Planel et al., 2007a). However, it is important to note that, even when inhibited, GSK-3β is still participating to Tau hyperphosphorylation, as we previously demonstrated with lithium, a GSK-3β inhibitor, in mice rendered hypothermic by starvation (Planel et al., 2001).

### Phosphatases inhibition

Ser/Thr protein phosphatases (PP) are classified into five types, PP1, PP2A, PP2B, PP2C, and PP5, on the basis of their substrate specificities and sensitivity to specific activators and inhibitors (Liu et al., 2005). Biochemical studies have demonstrated that PP1, PP2A, PP2B (Calcineurin), and PP5 are involved in Tau dephosphorylation (Gong et al., 1994; Tian and Wang, 2002). Importantly, it is believed that PP2A is the major Tau phosphatase *in vivo* (Gong et al., 2000; Planel et al., 2001, 2004, 2007a), with PP2A, PP1, PP5, and PP2B contributing to 71, 11, 10 and 7%, respectively, of the total Tau phosphatase activity in the brain (Liu et al., 2005).

The analysis of different Tau phosphatases in diabetic animal models is less documented in comparison to kinases (Table 1). However, although several investigators have observed a decrease only in the expression of PP2A in T2DM mice brains (Schubert et al., 2003; Li et al., 2012a), we (Planel et al., 2007b), and others (Clodfelder-Miller et al., 2006; Qu et al., 2011; Jung et al., 2013), have reported that PP2A activity is inhibited in both T1DM and T2DM animal models.

Interestingly, Deters and colleagues demonstrated that co-expression of P301L mutant human Tau and a dominant negative form of PP2A in the brain of transgenic mice significantly increases Tau hyperphosphorylation and NFT formation, suggesting a crucial role for PP2A in regulating Tau pathology (Deters et al., 2009). This finding is particularly important since PP2A is inhibited in human AD patients and seems to be an important factor in the progression of the disease (Gong et al., 1993, 1995; Vogelsberg-Ragaglia et al., 2001).

Thus, PP2A seems to be a key protein in the link between insulin dysfunction and AD. Extending analyses to other diabetic mouse models or genetically modified mice will facilitate the understanding of molecular mechanisms underlying PP2A function, and thus help for the identification of molecules that may compromise the reversibility of Tau hyperphosphorylation.

### Inflammation

Several studies have shown that insulin dysfunction is associated with inflammation. In the periphery, insulin was shown to modulate many aspects of the inflammatory process. Thus, at low doses, insulin exerts anti-inflammatory effects (Dandona, 2002); whereas, during chronic hyperinsulinemia, insulin may exacerbate inflammatory responses and increase markers of oxidative stress (Krogh-Madsen et al., 2004). Specifically, it was demonstrated that levels of proinflammatory cytokines, including Interleukin-1 (IL-1), IL-6, and C-reactive protein (CRP) are elevated in diabetic patients (Hak et al., 2001; Spranger et al., 2003).

In the CNS, several animal studies have also associated insulin dysfunction to inflammation. For example, a significant increase in the number of glial fibrillary acidic protein (GFAP)-reactive astrocytes (also known as astrogliosis) was reported in the hippocampi of both NOD and STZ-induced mouse models (Saravia et al., 2002).

In addition, T2DM was shown to aggravate vascular inflammation in an AD mouse model, changes that can be related to impaired central insulin signaling (Takeda et al., 2010). Notably, a study by Pistell et al. has established that different diet composition results in various inflammatory reactions in the brain. Thus, these authors have demonstrated that HFD consumption leads to a significant increase in the levels of proinflammatory cytokines, chemokines as well as reactive astrocytosis and microgliosis (Pistell et al., 2010). These data suggest that inflammation might be tightly related to Tau hyperphosphorylation observed during T2DM.

Moreover, brains of i.c.v.-STZ treated rats showed increased levels of IL-1 and tumor necrosis factor-α (TNF-α) (Chen et al., 2013a), as well as pronounced astrogliosis and microglial activation (Prickaerts et al., 1999; Chen et al., 2013a).

On the other hand, there is evidence that Tau pathology is associated with neuroinflammation. Thus, IL-1, IL-6 as well as nitric oxide have been shown to exacerbate Tau pathology and NFT formation *in vitro* (Li et al., 2003; Quintanilla et al., 2004; Saez et al., 2004). Moreover, Shepherd et al. have demonstrated that microglial activation might contribute to Tau hyperphosphorylation and NFT formation in *postmortem* AD brains (Shepherd et al., 2000; for review, see Arnaud et al., 2006). Similarly, administration of lipopolysaccharide (LPS), which is a bacterial endotoxin commonly used in animal studies to induce systemic inflammation (Lien et al., 2000), significantly increases Tau hyperphosphorylation in a triple transgenic mouse model of AD (Kitazawa et al., 2005). In addition, Yoshiyama et al. have demonstrated that microglial neuroinflammation precedes Tau pathology in P301S transgenic mice (Yoshiyama et al., 2007).

Mechanisms by which neuroinflammation might contribute to Tau pathology are not well elucidated. However, it is known that one signaling pathway by which neurons and microglia communicate is fractalkine (CX3CL1) and its cognate receptor (CX3CR1). In the CNS, CX3CL1 is highly expressed in neurons, whereas CX3CR1 is exclusively expressed in microglia cells (Harrison et al., 1998). Therefore, Bhaskar et al. have suggested that the CX3CL1/CX3CR1 pathway could modulate Tau pathology, and that this effect might be dependent upon microglial-derived IL-1-p38 MAPK signaling pathway (Bhaskar et al., 2010).

Overall, the link between insulin dysfunction, inflammation (both peripheral and/or central), and Tau hyperphosphorylation is unclear and would benefit from future research focus.

### Stress

Stress might represent another mechanistic link between insulin dysfunction and Tau hyperphosphorylation. Indeed, it was recently reported that stress leads to both peripheral and central insulin resistance, as well as increased Tau hyperphosphorylation in the mouse brain. Notably, these authors have pointed a central role of JNK (c-Jun N-Terminal Kinase), a major stress signaling pathway (Solas et al., 2013). These results suggest that insulin resistance might mediate Tau phosphorylation through a stress-dependent mechanism. Further studies are required to better understand this issue.

### Insulin signaling: the converging road

#### Insulin signaling impairment in diabetic animal models

While impaired peripheral insulin signaling is well known in animal models of diabetes, there is conflicting evidence of central insulin signaling dysfunction.

The phosphorylation levels of IRs were reported to be significantly decreased in mice treated with systemic STZ (Jolivalt et al., 2008, 2010), as well as in the insulin KO mouse model (Schubert et al., 2004) (Table 1). By contrast, levels of the β subunit of IR (IRβ) have shown contradictory findings; whereas Jung *et al* have observed that IRβ levels are increased in the brain of the OLETF T2DM rat model (Jung et al., 2011), Li et al. have reported a significant decrease in IRβ levels in the cortex of spontaneous T1DM, but not T2DM rats (Li et al., 2007). Similarly, the insulin KO mouse model has revealed a decrease in the expression of IRβ in the brain (Schechter et al., 2005).

Conflicting results have also been reported for Akt, a key kinase implicated in the insulin-signaling pathway, and also known to phosphorylate Tau *in vitro* (Ksiezak-Reding et al., 2003). Thus, a significant reduction in the phosphorylation levels and the activity of Akt (Li et al., 2007; Qu et al., 2011) was reported in the brain of both T1DM and T2DM animal models (Jolivalt et al., 2008, 2010; Qu et al., 2011; Bhat and Thirumangalakudi, 2013). By contrast, several data showed augmented phosphorylation of Akt and GSK-3β Ser9 (inhibitory phosphorylation) in NOD and STZ-treated mice (Clodfelder-Miller et al., 2005, 2006; Planel et al., 2007b). It is interesting to note that the rise in Akt and GSK-3β Ser9 phosphorylation in both NOD and STZ-treated mice is probably due to the inhibition of PP2A, as inhibitors of the phosphatase upregulate the phosphorylation of the two kinases (Andjelkovic et al., 1996; Planel et al., 2001). However, how peripheral insulin dysfunction results in central PP2A inhibition remains to be elucidated.

Moreover, Moroz et al. have demonstrated that HFD-induced T2DM leads to an increase in mRNA levels of IRS-1 and IRS-4 in the brain of non-transgenic mice (Moroz et al., 2008). By contrast, Bhat and Thirumangalakudi have reported a decrease in IRS-1 levels in the brain of HFC-treated mice (Bhat and Thirumangalakudi, 2013).

Interestingly, a recent study has demonstrated that HFD potentiated Tau pathology in a mouse model of Tauopathy, in a manner independent from insulin resistance, suggesting that other factors, probably linked to obesity, might be implicated in Tau pathology during T2DM (Leboucher et al., 2012).

Several investigators have also reported a deregulation in gene and/or protein expression of the insulin-signaling pathway in T3DM animal models. For example, the phosphorylation level of PI3K was significantly decreased in mice treated with central STZ (1.5 mg/Kg, i.c.v.) (Deng et al., 2009). Moreover, Grunblatt et al. have demonstrated that mRNA levels of both insulin and IRs are decreased, whereas levels of phosphorylated IRβ are increased in the hippocampi of T3DM rats (Grunblatt et al., 2007), (Table 1).

In summary, the involvement of the central insulin signaling in Tau pathology is still controversial, with divergent results showing a decrease, an increase, or even no significant changes in the insulin signaling proteins in the brains of diabetic animals (Table 1). Therefore, further investigations are crucial to get a more definitive picture of the role of central insulin signaling in the progression of Tau pathology.

#### Implication of insulin signaling on Tau pathology

Beside its classical function of regulating glucose metabolism, there is increasing evidence supporting a role for the insulin-signaling pathway in neuronal development as well as in learning and memory, therefore suggesting a crucial role of insulin signaling in AD pathogenesis (Zhao and Alkon, 2001; Craft and Watson, 2004; Gerozissis, 2008).

It has been proposed that insulin could affect Tau phosphorylation through the regulation of GSK-3β, since it is a kinase downstream in the IR signaling pathway (Hong and Lee, 1997). Although insulin administration in STZ treated mice completely (Planel et al., 2007b), or partially (Clodfelder-Miller et al., 2006; Jolivalt et al., 2008) rescued Tau phosphorylation in mice, these findings are in contrast with human studies that report a higher risk of AD in patients treated with insulin (Ott et al., 1999; Luchsinger et al., 2001). However, one should be cautious in making direct conclusions from these epidemiological studies, as it might be because the patients were having a more severe stage of diabetes that they required treatment with insulin. Thus, more controlled studies in animals, as well as in human patients are important to better understand the role of insulin in the brain.

Furthermore, several studies have investigated the impact of rosiglitazone treatment on Tau phosphorylation in animal models. For example, Escribano et al. have observed a significant decrease in Tau phosphorylation in the brain of transgenic hAPP mice following rosiglitazone treatment (Escribano et al., 2010). Similarly, Yoon et al. have demonstrated that this drug reduced Tau phosphorylation both in *vitro*, and in the hippocampi of OLETF T2DM rats. Interestingly, these authors suggest that this effect might be due to a decrease in JNK activity (Yoon et al., 2010).

Thus, although brain insulin signaling might have an important effect on Tau phosphorylation, the molecular mechanisms underlying this effect are far from clear. Better understanding of these mechanisms might help to develop therapeutic strategies aiming to reduce Tau hyperphosphorylation in the brain.

## Conclusion

**Figure 1:**
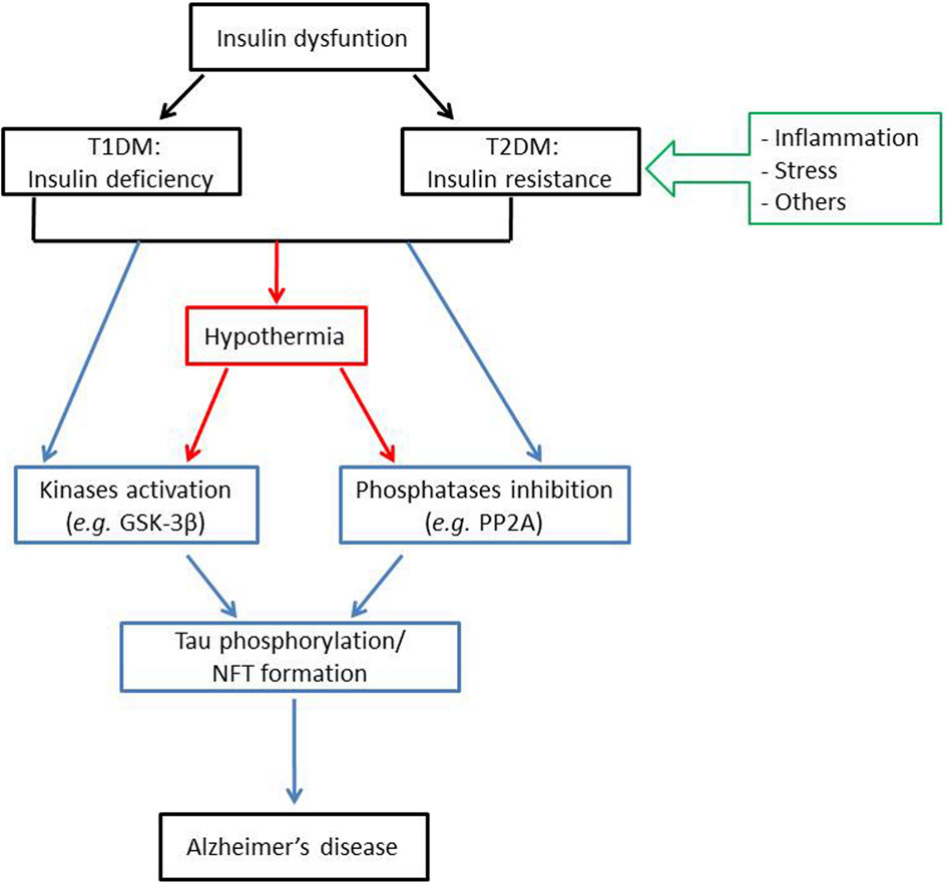
**Insulin dysfunction might enhance Alzheimer's disease pathology through distinct mechanisms**. Insulin dysfunction, manifested by either T1DM, characterized by insulin deficiency; or T2DM, characterized by insulin resistance, might lead to Tau phosphorylation and NFT formation, one of the major histopathological hallmarks of Alzheimer's disease. This effect can be induced either directly, through kinases activation (such as GSK-3β) and/or phosphatases inhibition (such as PP2A); or indirectly, through the effect of hypothermia on kinases and phosphatases activities. For note, insulin resistance might be related to Tau pathology through several mechanisms, mainly including inflammation and stress. Abbreviations: T1DM: type 1 diabetes mellitus; T2DM: type 2 diabetes mellitus; GSK-3β: glycogen synthase kinase 3β; PP2A: protein phosphatase 2A; NFT: neurofibrillary tangles.

Overall, numerous preclinical studies examining the correlation between insulin dysfunction and Tau hyperphosphorylation converge to indicate that both T1DM and T2DM might affect Tau pathology, either directly or indirectly (Figure 1). Apart from some emerging evidences, mechanisms by which insulin dysfunction and/or other features of diabetes such as obesity and inflammation contributes to Tau pathology are still not fully elucidated. Therefore, further studies using other animal models (e.g., ob/ob mice…) are required to better understand the contributions of these mechanisms to Tau hyperphosphorylation. However, future studies must carefully report and control physiological parameters such as body temperature, and should avoid using anesthesia for the sacrifice of the animals.

In light of the increased incidence of diabetes in the young population, future focus on the correlation between insulin dysfunction and Tau pathology may provide invaluable information for the treatment and prevention of sporadic AD.

### Conflict of interest statement

The authors declare that the research was conducted in the absence of any commercial or financial relationships that could be construed as a potential conflict of interest.
